# Disease-Causing Mutations and Rearrangements in Long Non-coding RNA Gene Loci

**DOI:** 10.3389/fgene.2020.527484

**Published:** 2020-11-30

**Authors:** Marina Aznaourova, Nils Schmerer, Bernd Schmeck, Leon N. Schulte

**Affiliations:** ^1^ Institute for Lung Research, Philipps University Marburg, Marburg, Germany; ^2^ Systems Biology Platform, German Center for Lung Research (DZL), Philipps University Marburg, Marburg, Germany; ^3^ Center for Synthetic Microbiology (SYNMIKRO), Philipps University Marburg, Marburg, Germany

**Keywords:** long non-coding RNA, genome-wide association study, variation (genetic), disease, single nucleotide polymorphism, mutation

## Abstract

The classic understanding of molecular disease-mechanisms is largely based on protein-centric models. During the past decade however, genetic studies have identified numerous disease-loci in the human genome that do not encode proteins. Such non-coding DNA variants increasingly gain attention in diagnostics and personalized medicine. Of particular interest are long non-coding RNA (lncRNA) genes, which generate transcripts longer than 200 nucleotides that are not translated into proteins. While most of the estimated ~20,000 lncRNAs currently remain of unknown function, a growing number of genetic studies link lncRNA gene aberrations with the development of human diseases, including diabetes, AIDS, inflammatory bowel disease, or cancer. This suggests that the protein-centric view of human diseases does not capture the full complexity of molecular patho-mechanisms, with important consequences for molecular diagnostics and therapy. This review illustrates well-documented lncRNA gene aberrations causatively linked to human diseases and discusses potential lessons for molecular disease models, diagnostics, and therapy.

## Introduction

**Figure 1:**
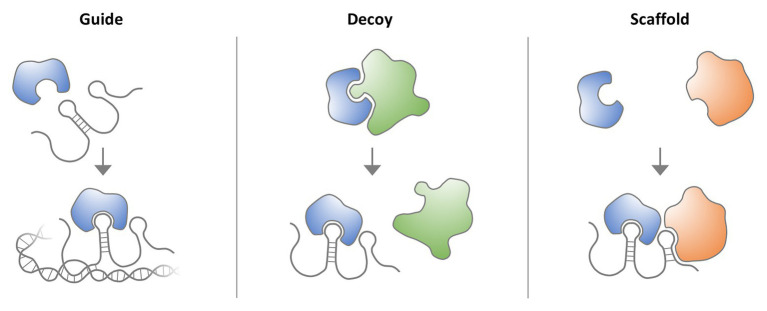
Molecular lncRNA mechanisms. LncRNAs interact with proteins, such as transcription factors, signaling complexes or enzymes to regulate their activity. LncRNAs can act as guides, to, for example, direct transcription factors to DNA binding sites. LncRNAs can also act as decoys to block the binding of a protein to other proteins or nucleic acids. Finally, lncRNAs can serve as scaffolds for the assembly of multi-protein complexes.

An important lesson from the decryption of the human genome sequence and the subsequent systematic mapping of transcribed gene regions is that a surprisingly small proportion of the genome (~2%) encodes proteins. Rather, most of the genome sequence consists of regulatory and structural DNA regions, which harbor thousands of non-coding RNA (ncRNA) loci, including long non-coding RNA (lncRNA) genes (Derrien et al., 2012). LncRNAs are defined as transcripts ≥200 nucleotides in length, which do not encode proteins (Derrien et al., 2012) but may regulate the activity of proteins, such as transcription factors or enzymes, by functioning as guides, decoys, or scaffolds (Figure 1). LncRNAs can, for instance, act at the chromatin level by forming RNA-DNA hybrids at enhancer and promoter elements and by associating with transcription factors (Rinn and Chang, 2012). Besides guiding and decoying chromatin-regulatory protein complexes, such as the polycomb repressor complexes (PRCs), lncRNAs can also mediate the tethering of distal enhancer elements to gene promoters. Disruption of lncRNA functions consequently contributes to diseases by disturbing key transcriptional circuitries, such as the control of MYC oncogene expression through lncRNA CCAT1-L-associated enhancers (Xiang et al., 2014). Of note, besides regulatory RNA-protein interactions, the act of non-coding RNA transcription itself can adopt important cellular functions (Ali and Grote, 2020). This is exemplified by the prevention of DNA methylation at CTCF binding sites by RNA polymerase II (RNAPolII) mediated ThymoD ncRNA transcription, resulting in a chromatin loop fostering Bcl11b enhancer-promoter interaction (Isoda et al., 2017; section Discussion). Besides the well-established functions in the nucleus, lncRNAs are increasingly recognized to act in cytoplasmic circuitries, where they e.g., regulate translation or mitochondrial function (Carlevaro-Fita and Johnson, 2019). Whereas only few functional lncRNA loci had been discovered until the late 2000s, systematic annotation efforts by the ENCODE and FANTOM consortia have revealed thousands of human lncRNA loci (Derrien et al., 2012; Hon et al., 2017) and numerous recent studies suggest that lncRNAs are critically involved in many human diseases, including Type II diabetes, cancer, or AIDS (Huarte, 2015; Lazar et al., 2016; He et al., 2017). This suggests that the prevalent protein-centric understanding of human diseases does not capture the full complexity of molecular patho-mechanisms, with important consequences for patient genotyping and personalized therapy approaches.

**Figure 2:**
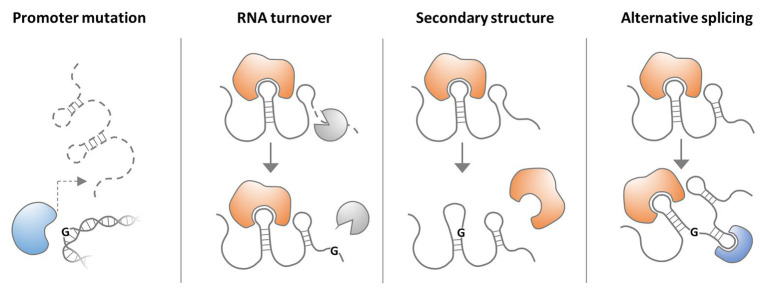
Possible consequences of lncRNA single nucleotide polymorphisms (SNPs). A SNP in an lncRNA gene promoter may interfere with transcription factor binding and thus lncRNA expression. A SNP in the lncRNA sequence may affect RNA-turnover by altering the binding of proteins regulating lncRNA stability. An lncRNA SNP may also alter RNA secondary structure and thus impact on binding of protein partners. Finally lncRNA SNPs may affect splice sites and thereby alter transcript architecture and interaction with proteins.

While lncRNA research is still at its infancy, many genome-wide association studies (GWASs) have implicated lncRNA gene polymorphisms in human diseases. The vast majority of GWAS-identified single nucleotide polymorphisms (SNPs) map to intergenic and intronic sequences (Hindorff et al., 2009), supporting the notion that the non-coding regions of the genome are critically involved in disease predisposition. Non-coding SNPs were found to be particularly enriched in promoters, including DNaseI hypersensitive sites (HSSs; Hindorff et al., 2009; Maurano et al., 2012), which represent hallmarks of active transcription and are required for lncRNA gene expression (Janga et al., 2018). Consequently, recent evidence supports the concept that SNPs may affect the expression of disease-associated lncRNAs through alteration of regulatory DNA regions, such as transcription factor binding sites (Figure 2; Kulkarni et al., 2019). Other consequences of nucleotide rearrangements in disease-relevant lncRNA loci, such as altered secondary structures or alternative splicing, are conceivable (Figure 2). Besides SNPs, long-range alterations, such as amplifications, deletions, and translocations were reported to cause diseases through lncRNA copy number alterations (CNAs) and by affecting lncRNA integrity. Furthermore, several lncRNAs with disease-associated gene variants were proposed as clinical markers, facilitating e.g., stratification and survival prognosis of cancer patients (Zhang et al., 2017c; Arriaga-Canon et al., 2018).

Despite the wealth of recently published lncRNA literature, including GWAS and prognostic marker studies, the *in vivo* relevance of lncRNAs in human diseases remains debated, due to contradictory results from cell culture, small animal, and clinical studies. MALAT1, for instance, is abundantly expressed in a variety of human cell types and was among the first lncRNAs to be mechanistically characterized. It is an important structural component of nuclear speckles and has been linked to cancer cell proliferation and metastasis (Zhang et al., 2017c). These results are contrasted by the lack of obvious phenotypes in MALAT1 deficient mice (Eissmann et al., 2012). Whereas cancer-associated polymorphisms have been recurrently identified within the MALAT1 gene locus (Fujimoto et al., 2016; Rheinbay et al., 2020), recent studies cast doubt on the ability of MALAT1 polymorphism data, alone, to establish a causative relationship between the lncRNA and human disease (Carlevaro-Fita et al., 2020; Rheinbay et al., 2020). Additional inclusion of somatic mutation data from tumor genomics or evidence from transposon-mutagenesis screens in mice may pinpoint cancer-driver lncRNAs, including e.g., NEAT1 (Carlevaro-Fita et al., 2020). Presently, however, only few such well-curated resources and meta-analysis pointing out causal associations of lncRNA gene aberrations with diseases are available. Specialized databases, such as LincSNP 3.0, LncRNADisease 2.0, or NONCODE (Table 1), currently serve as the major knowledge hubs, listing hundreds of reported lncRNA disease associations and polymorphisms, reiteration of which would be beyond the scope of the present review. Rather, we aim at providing an overview of documented disease-causative human lncRNA gene abberations, which have been genetically characterized and independently confirmed or mechanistically dissected (Table 2). We discuss the utility of such knowledge in molecular diagnostics, patient stratification, and prospective personalized therapy approaches.

**Table 1:** Databases listing long non-coding RNA (lncRNA) gene variants and disease-associations.

Database	Description and URL	Reference
LincSNP	Disease and phenotype associated gene variants. http://bio-bigdata.hrbmu.edu.cn/lincsnp/index.jsp	Ning et al., 2017
LncVar	SNPs, eQTLs, and copy number variations. http://159.226.118.31/LncVar/	Chen et al., 2017
LncRNADisease	Experimentally supported and predicted disease associations. http://www.rnanut.net/lncrnadisease/index.php/home	Bao et al., 2019
EVLncRNAs	Experimentally validated disease associations. http://biophy.dzu.edu.cn/EVLncRNAs/	Zhou et al., 2018
CLC	List of lncRNAs causally implicated in cancer. https://www.gold-lab.org/clc	Carlevaro-Fita et al., 2020
Lnc2Cancer	Experimentally supported associations with cancer. http://www.bio-bigdata.com/lnc2cancer/	Gao et al., 2019
LncBook	Knowledge database (includes disease-associations and SNPs). https://bigd.big.ac.cn/lncbook/index	Ma et al., 2019
NONCODE	Knowledge database (includes disease-associations and SNPs). http://www.noncode.org/	Fang et al., 2018
LncRNAWiki	Platform for community curation of lncRNA information (including disease associations). http://lncrna.big.ac.cn/index.php/Main_Page	Ma et al., 2015

**Table 2:** LncRNAs covered in the present review, genetically linked to human diseases.

LncRNA	Mechanism	Disease	Reference
ALAL-1	*trans* Interacts with SART3 and regulates USP4 dequbiquitinase subcellular shuttling	Adenocarcinoma	Athie et al., 2020
ANRIL	*cis* and *trans* Regulation of CDKN2A/B *via* binding to CBX7, interaction with YY1 transcription factor	Melanoma, breast cancer, neuronal system tumor, type 2 diabetes, glaucoma, cardiovascular diseases	Helgadottir et al., 2007; McPherson et al., 2007; Pasmant et al., 2007; Scott et al., 2007; Zeggini et al., 2007; Broadbent et al., 2008; Stacey et al., 2009; Bei et al., 2010
ATXN80S	?	Spinocerebellar ataxia, Parkinson disease	Koob et al., 1999; Samukawa et al., 2019
CCAT2	*trans* Induction of expression of WNT signaling genes, regulation of MYC expression, and alternative splicing of GLS mRNA	Cancer	Pomerantz et al., 2009; Ling et al., 2013; Redis et al., 2016; Xin et al., 2017
CCR5AS	*cis* Enhancement of CCR5 mRNA stability *via* suppressive interaction with RALY	HIV infection	McLaren et al., 2015; Kulkarni et al., 2019
CUPID1/2	*trans* Locate to nuclear foci and promote DNA repair by homologous recombination	Breast cancer	Betts et al., 2017
DA125942	*cis* PTHLH expression	BDE	Maass et al., 2012
DBE-T	*cis* Recruitment of Ash1L to FSHD gene locus	FSHD	Cabianca et al., 2012
DIRC3	*trans* promotes expression of the tumor suppressor IGFBP5	Renal carcinoma, melanoma	Bodmer et al., 2003; Coe et al., 2019
DISC2	?	Schizophrenia	Millar et al., 2000; Blackwood et al., 2001; Chubb et al., 2008
FAL1	*trans* Stabilization of BMI1	Ovarian cancer, prostate cancer	Hu et al., 2014
Gas5	*cis* and *trans* Fusion with BCL6, decoy of the glucocorticoid receptor	B-cell lymphoma	Nakamura et al., 2008
HOTAIR	*trans* Regulation of HOX gene expression	Cancer	Rinn et al., 2007; Tang and Hann, 2018
HULC	*trans* Interaction with YB1, coREST, and hnRNPK	Cancer, hepatocellular carcinoma, colorectal cancer, esophageal cancer	Liu et al., 2012; Kang et al., 2015; Shaker et al., 2017; Yu et al., 2017; Chen et al., 2019b
H19	*trans* Interaction with chromatin factors let-7 microRNA sponge	Atherosclerosis, coronary artery disease, predisposition to elevated blood-pressure, cancer	Han et al., 1996; Verhaegh et al., 2008; Kallen et al., 2013; Monnier et al., 2013; Tragante et al., 2014; Gao et al., 2015; Sung et al., 2018; Li et al., 2018a
IPW	*trans* Reduction of gene expression in DLK1-DIO3 gene locus	PWS	Wevrick et al., 1994
IFNG-AS	*cis* Interaction with WDR5, promotion of IFNγ expression	IBD	Vigneau et al., 2003; Mirza et al., 2015; Padua et al., 2016
Lnc-NR2F1	*trans* Binds to chromatin and regulates neuronal genes	Developmental disorders	Ang et al., 2019
Lnc13	*cis* and *trans* Repression of inflammatory gene transcription *via* interaction with hnRNPD	Celiac disease	Castellanos-Rubio et al., 2016
LINC00237	?	MOMO syndrome	Vu et al., 2012
LINC00305	?	Atherosclerosis, rheumatoid arthritis	Zhang et al., 2017b; Wahba et al., 2020
Linc-HELLP	*trans* Alteration of protein interactions involved in splicing and ribosomal function	HELLP syndrome	van Dijk et al., 2012, 2015
LOC285194	?	Osteosarcoma	Li et al., 2017b; Pasic et al., 2010
MIAT	*trans* Interacts with the nuclear matrix and associates with splice factor SF1	Cardiovascular diseases	Ishii et al., 2006; Sone et al., 2007; Tsuiji et al., 2011; Ishizuka et al., 2014
MIR2052HG	*trans* Promotes ERα expression and stability	Breast cancer	Ingle et al., 2016
NEAT1	?	Cancer, breast cancer, neural degeneration	Fujimoto et al., 2016; Atianand et al., 2017; Rheinbay et al., 2017; An et al., 2018; Li et al., 2018b
OVAL	?	Ovarian adenocarcinoma and endometrial carcinoma	Akrami et al., 2013
PCAN-R1 and PCAN-R2	?	Prostate cancer	Du et al., 2013
PCAT1	*trans* Regulation of androgen-stimulated genes *via* interaction with AR/LSD1 complex	Prostate cancer	Prensner et al., 2011
PRAL	*trans* Regulates p53 stability	hepatocellular carcinoma	Zhou et al., 2016a
PTCSC3	?	papillary thyroid carcinoma	Jendrzejewski et al., 2012
PVT1	*cis* and *trans* Stabilization of MYC, KLF5 and STAT3 Fusion with CHD7	Cancer, small cell lung carcinoma	Pleasance et al., 2010; Tseng et al., 2014; Tang et al., 2018; Zhao et al., 2018; Jin et al., 2019
RMRP	*trans* rRNA maturation?	CHH, cancer	Martin and Li, 2007; Zack et al., 2013; Rheinbay et al., 2020
RMST	*trans* Associates with hnRNPA2/B1 and SOX2 to promote SOX2 target gene expression	Kallmann syndrome	Ng et al., 2013; Stamou et al., 2020
RUNXOR	*trans* engaged in chromatin loops, relevant to RUNX1 translocations	Acute myeloid leukemia	Wang et al., 2014
SAMMSON	*trans* Regulation of mitochondrial metabolism *via* interaction with p32	Melanoma	Leucci et al., 2016
SNHG17	?	Cancer	Ma et al., 2017; Xu et al., 2019

## Disease-Causing Genomic Rearrangements Affecting IncRNA Loci

Genomic rearrangements, such as deletions, amplifications, or translocations may alter gene expression, e.g., through the deletion of regulatory DNA elements, or the fusion of silent genes to active enhancer or promoter regions. Such aberrations are key events in the development of prevalent diseases such as cancer, but also in rare Mendelian disorders. Of note, genomic rearrangements causing disease (driver events) need to be discriminated from passenger rearrangements, which constitute a consequence rather than the cause of a disease. Ambitious whole genome sequencing (WGS) projects, such as the 100,000 genome consortiums, and the advent of WGS in the clinics promise to systematically uncover such disease-promoting genomic alterations and to transform molecular diagnosis and patient stratification (Siva, 2015; Nik-Zainal et al., 2020). So far, however, genome-based diagnostics predominantly focuses on protein-coding genes, and the consequences of lncRNA gene disruptions are only beginning to be appreciated. Several studies have implicated deletions, amplifications, and translocations within lncRNA loci in diseases, such as cancer, schizophrenia, or muscular dystrophy. Section Disease-Causing Genomic Rearrangements Affecting lncRNA Loci summarizes lncRNA gene rearrangements causatively linked to human diseases. Disease-associated amplifications, deletions, and translocations affecting lncRNA genes are complemented by a plethora of SNPs, many of which have been causally linked to disease manifestation and are covered in section SNPs in lncRNA Loci Causally Implicated in Disease.

### Amplifications and Deletions

**Figure 3:**
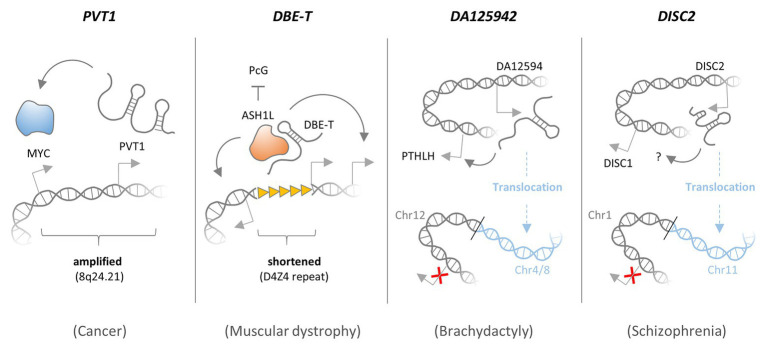
Functional consequences of amplifications, deletions, and translocations affecting lncRNAs. The PVT1 lncRNA gene locates to a fragile region on chromosome 8 (8q24.21) and is co-amplified with the MYC proto-oncogene in many types of cancer. PVT1 expression is required for maintenance of high MYC protein levels, and thus PVT1 co-amplification promotes MYC activity and cancer cell proliferation. LncRNA DBE-T is generated preferentially in patients with facioscapulohumeral muscular dystrophy (FSHD), as a consequence of D4Z4 repeat deletions in the 4q35 region. In the presence of an intact D4Z4 repeat, Polycomb group (PcG) complexes bind and repress local transctiption. When the D4Z4 repeat is shortened, DBE-T recruits the protein Ash1L, to promote transcription of neighboring genes in the FSHD locus, thereby promoting the disease. DA125942 is an lncRNA encoded on chromosome 12, which binds to and thereby promotes transcription of the *PTHLH* gene in cis. A balanced translocation removes the *DA12594* gene from chromosome 12 and thereby blunts PTHLH transcription, which causes Brachydactyly. Disrupted in schizophrenia 2 (DISC2) is an lncRNA encoded on chromosome 1, promoting expression of the neighboring DISC1 gene in cis through a yet unknown mechanism. A balanced translocation removes the DISC2 gene from chromosome 1 and blunts DISC1 expression, which may cause Schizophrenia.

Deletions and amplifications may occur through chromosomal crossover and repair events or replication defects in somatic cells and in the germ-line. While severe germ-line gene defects may stall fetal development (Kacprzak et al., 2016), deletion, or amplification of regulatory DNA elements in proximity to critical genes such as cell-division checkpoint genes may alter their transcript output and predispose to diseases such as cancer. Besides inheritable predispositions, somatically acquired defects in cell division and differentiation genes, e.g., through UV-light exposure or inhalation of mutagens like cigarette smoke, are major drivers of cancer. The molecular diagnostic identification of causative somatic CNAs (SCNAs) due to amplifications or deletions may help to determine appropriate treatment regimens (Nik-Zainal et al., 2020). Recently, besides classic protein-coding genes, such as *MYC*, *RB*, or *p53*, amplifications and deletions affecting lncRNA loci were shown to contribute to oncogenesis (Figure 3). For instance, germ-line deletions of lncRNA ANRIL, located in the CDKN2A/B or INK4-ARF locus on chromosome arm 9p21, have been associated with an increased risk of melanoma and neural system tumor development in several families in the United States and Europe (Bahuau et al., 1998; Pasmant et al., 2007). Multiple GWAS studies have confirmed a role of ANRIL in cancer and other diseases (section SNPs Affecting lncRNA Genes Implicated in Oncogenesis). Meanwhile, further lncRNA disruptions due to amplifications and deletions causatively linked to cancer and other diseases have been identified.

#### NEAT1

The essential nuclear speckle component NEAT1 belongs to the most well-studied lncRNAs. Besides its role as an architectural RNA of nuclear bodies, NEAT1 is also involved in inflammatory, neurodegenerative, and other disease-associated cellular processes (Atianand et al., 2017; An et al., 2018). Furthermore, NEAT1 is over-expressed in many solid tumors (Li et al., 2018b) and knockdown of NEAT1 decreased cell invasion (Fujimoto et al., 2016). Multiple recent reports suggest a causal link between NEAT1 gene aberrations and cancer. NEAT1 was identified as one out of three genes with recurrent mutations in breast cancer. Interestingly, three out of four identified promoter mutations interfered with NEAT1 expression, in line with its frequent focal deletion in breast cancers (Rheinbay et al., 2017). Thus, both NEAT1 gain- and loss-of-function might contribute to oncogenesis. In a pan-cancer study, an enrichment of indel mutations in the NEAT1 locus was observed. It was doubted, however, that these constitute driver mutations (Rheinbay et al., 2020). In another recent study, including information from transposon-mutagenesis screens, however, NEAT1 was identified as one out of eight lncRNAs with cancer driver mutations (Carlevaro-Fita et al., 2020). Different from other well-studied lncRNAs, such as PVT1 or ANRIL (see below), the precise molecular mechanisms by which NEAT1 impacts on oncogenesis remain to be determined. Taken together, the well-studied RNA component of nuclear speckles, NEAT1, is recurrently mutated and subjected to SCNAs, causally implicated in cancer.

#### PVT1

8q24.21 is a fragile region on chromosome 8, which is amplified in many types of cancer and harbors the MYC proto-oncogene (Jin et al., 2019). Increased MYC expression has been implicated in the development of many types of tumors. Consequently, protective cellular mechanisms exist, which induce growth arrest or cell death in response to elevated MYC expression (Gabay et al., 2014). Thus, MYC activation alone is typically not sufficient to reinforce cell transformation programs and initiate tumorigenesis. Besides the MYC gene, the 8q24.21 region harbors the PVT1 lncRNA locus, expression of which is consequently elevated in various types of cancer tissues and cell lines as a result of amplifications and translocations (Boloix et al., 2019; Jin et al., 2019). A report by Tseng et al. (2014) suggests that the gain of PVT1 lncRNA expression is required for the maintenance of high MYC protein levels and cancer cell proliferation. Besides MYC, PVT1 was also shown to stabilize other cell proliferation and survival promoting proteins, such as KLF5 and STAT3 (Tang et al., 2018; Zhao et al., 2018). Consequently, PVT1 was identified as a prognostic marker for cancer patient’s overall survival, and elevated PVT1 expression was found to associate with advanced tumor severity stage according to the tumor-node-metastasis (TNM) classification scheme (Lu et al., 2017; Martini et al., 2017; He et al., 2018; Boloix et al., 2019). Interestingly, two alternative PVT1 transcriptional start sites were shown to serve as enhancer elements, directly regulating MYC gene expression independent of PVT1 lncRNA expression (Fulco et al., 2016). Thus, the PVT1 lncRNA locus, which is often co-amplified with the MYC gene, may contribute to tumorigenesis through both lncRNA-mediated MYC protein stabilization and an lncRNA independent enhancer function (Figure 3).

#### FAL1

An array- and shRNA-based screen across tumor tissues from various types of cancer pinpointed FAL1 as an oncogenic lncRNA expressed from a focal amplicon on chromosome 1q21.2 (Hu et al., 2014). FAL1 copy number gain significantly associated with decreased ovarian cancer and prostate cancer patient’s overall survival (Hu et al., 2014; Zhao et al., 2017). In melanoma patients, FAL1 expression was shown to have prognostic value with respect to lymph node metastasis and TNM stage, as well as overall survival (Ni et al., 2017). Altered expression and prognostic potential of FAL1 have also been reported in the context of diverse other types of cancer (Lv et al., 2019). Mechanistically, FAL1 associates with and stabilizes the PRC 1 component BMI1 in a ubiquitin-proteasome dependent manner to suppress expression of the cyclin-dependent kinase inhibitor p21 (Hu et al., 2014). Thus, similar to NEAT1 and PVT1, CNAs affecting lncRNA FAL1 are causally involved in oncogenesis and have prognostic potential.

#### RMRP

Long non-coding RNA RMRP is a component of the mitochondrial RNA processing endoribonuclease MRP, which is involved in rRNA maturation (Martin and Li, 2007). Germ-line point-mutations, insertions, and duplications reducing RMRP expression cause the recessively inherited disorder “cartilage-hair hypoplasia” (CHH), which is primarily associated with abnormalities in bone and cartilage development (Ridanpaa et al., 2001; Martin and Li, 2007; Nakashima et al., 2007). Besides CHH, several cancer-genome studies have revealed RMRP SCNAs due to focal amplifications (Zack et al., 2013; Rheinbay et al., 2020), and the RMRP promoter was found to contain a breast-cancer associated mutation hotspot, impacting RMRP transcription (Rheinbay et al., 2017). In tissue samples from other types of cancer, RMRP mutations were identified with only low prevalence (0–1.3%), suggesting that RMRP SCNAs do not constitute generalized cancer drivers or biomarkers, beyond the well-documented implications in breast cancer (Son et al., 2019). Thus, loss and gain of lncRNA RMRP expression due to point-mutations and amplifications is causally linked to CHH and breast cancer, respectively.

#### SAMMSON

Similar to RMRP, SAMMSON is a nuclear encoded lncRNA localized to mitochondria. It promotes mitochondrial metabolism by fostering mitochondrial localization of rRNA processing factor p32 (Leucci et al., 2016). In melanoma patients, the SAMMSON locus is recurrently co-gained with the MITF oncogene through 3p13-3p14 focal amplification. Additionally, SAMMSON expression in melanoma cells is promoted by the SOX10 transcription factor. SAMMSON promotes melanoma cell viability and desensitizes the cells to MAPK-targeting cancer therapeutics (Leucci et al., 2016). Thus, both RMRP and SAMMSON are mitochondrial metabolic regulators with recurrent SCNAs due to focal amplifications, causally involved in cancer. Of note, MITF, with which SAMMSON is co-amplified at 3p13-3p14, is a transcriptional regulator of lncRNA DIRC3, which is disrupted by balanced translocations in renal carcinoma and suppressed by SOX10 in melanoma cells (see Translocations).

#### DBE-T

Facioscapulohumeral muscular dystrophy (FSHD) is an inherited disease, which is characterized by progressive skeletal muscle weakness. The disease has been linked to a shortened D4Z4 repeat in the subtelomeric chromosome region 4q35, which controls expression of the FSHD gene locus in cis (Sacconi et al., 2015). The FSHD locus may be silenced by PRC proteins, which bind to the D4Z4 repeat and establish repressive chromatin marks, or activated by Trithorax proteins during myoblast differentiation (Bodega et al., 2009; Cabianca et al., 2012). In FSHD patients, shortening of the D4Z4 repeat region was found to activate the FSHD locus through an epigenetic mechanism involving the cis-acting lncRNA DBE-T. DBE-T is generated preferentially in FSHD patients, as a consequence of D4Z4 repeat deletions and recruits the Trithorax protein Ash1L, to promote H3K36 dimethylation and transcription of the FSHD locus (Cabianca et al., 2012; Figure 3). Thus, increased expression of nuclear lncRNA DBE-T due to D4Z4 repeat shortening induces chromatin-changes at the site of lncRNA transcription, triggering expression of neighboring disease-associated transcripts.

#### IPW

Imprinting controls the exclusive expression of genes from the paternal or maternal chromosome. Microdeletions in the imprinted 15q11-q13 region on the paternal chromosome cause Prader-Willi Syndrome (PWS), a genetic disease associated with life-threatening obesity, but also learning and behavioral problems (Butler et al., 2019). Already in 1994, the PWS-associated chromosomal region was shown to give rise to the IPW lncRNA, which is lost in PWS patients (Wevrick et al., 1994). Encoded on chromosome 15, IPW acts in *trans* to reduce expression of genes from the DLK1-DIO3 locus on maternal chromosome 14. Thus, loss of trans-regulation of DLK1-DIO3 expression by IPW might contribute to PWS (Stelzer et al., 2014). IPW spans the SNORD116 snoRNA cluster, and deletions affecting these snoRNAs were implicated in PWS in humans (Sahoo et al., 2008). In mice, the lncRNA host gene giving rise to SNORD116 snoRNAs was shown to interact with transcriptional regulator RBBP5 in the nucleus. Deletion of the murine SNORD116 host-gene disrupted expression of circadian clock genes and caused altered diurnal energy expenditure in the brain, which might explain some of the neurological problems in PWS patients (Powell et al., 2013). Taken together, clinical and genetic evidence collected over the past 25 years pinpoints disruption of the 15q11-q13 region as a common cause of PWS, rendering it one of the first lncRNA loci linked to a human genetic disease. The precise mechanistic contributions of 15q11-q13 encoded ncRNAs to PWS still remain to be determined.

Besides the well-documented amplifications and deletions illustrated above, several other lncRNA CNAs have been reported and linked to human diseases. For instance, an expanding tri-nucleotide repeat within the ATXN8OS lncRNA locus silences ATXN8OS expression and has been associated with spinocerebellar ataxia and Parkinson disease (Koob et al., 1999; Samukawa et al., 2019). ALAL-1, an immune-inducible lncRNA, is amplified in adenocarcinomas and promotes tumorigenesis (Athie et al., 2020). LncRNA LOC285194 is recurrently deleted in osteosarcoma and associated with poor survival (Pasic et al., 2010; Li et al., 2017b). Focal amplification of the OVAL lncRNA locus on chromosome 1 was observed in ovarian adenocarcinoma and endometrial carcinoma (Akrami et al., 2013). In a screen for cancer driver lncRNAs, PCAN-R1 and PCAN-R2 were identified, which locate to SCNA regions and display increased expression in prostate cancer tissue. Knockdown of both lncRNAs slowed down prostate cancer cell growth *in vitro* (Du et al., 2013). Similarly, SNHG17, which is overexpressed in different cancer tissues, likely through amplifications, promotes cancer cell proliferation and constitutes a marker of poor prognosis (Ma et al., 2017; Xu et al., 2019). PRAL is a recurrently deleted lncRNA in hepatocellular carcinoma, which regulates p53 stability (Zhou et al., 2016a). Additional, lncRNAs are likely subjected to CNAs and constitute potential cancer drivers, as suggested by systematic screens (Zhou et al., 2017; Volders et al., 2018; Luo et al., 2019; Athie et al., 2020). While dozens of additional cancer-associated lncRNAs exist (Du et al., 2013; Carlevaro-Fita et al., 2020), studies systematically linking lncRNA CNAs due to amplifications and deletions to clinical outcomes, and disease mechanisms are still scarce. The examples illustrated above, however, suggest that further research into the clinical relevance of lncRNA CNAs may reveal additional driver mutations, valuable prognostic markers, and potential novel therapeutic angles.

### Translocations

Besides amplifications and deletions, translocations may affect the functionality and the transcriptional output of disease-relevant lncRNA genes. The term “translocation” describes an unusual chromosomal rearrangement, e.g., caused by a chromosomal break, which is followed by fusion of the resulting fragment to a different chromosome. Balanced translocations, in which no genetic material is lost, may occur without causing disease symptoms. Sometimes, however, the function of disease-relevant genes is altered. Translocations may occur non-randomly at chromosomal break-points, and many of the resulting malignancies have been characterized (Aplan, 2006). LncRNA genes (e.g., lnc-RP11-211G3.3.1-1 or FAM230C) may demarcate such chromosomal break-points (Lu et al., 2015; Delihas, 2018) or be fused to regulatory elements as a result of translocations, thereby causing disease. For instance, fusion of the 5' region of the PVT1 lncRNA gene to the CHD7 chromatin remodeler gene likely results in CHD7 gain-of-function in small cell lung cancer (Pleasance et al., 2010). Dozens of further cancer-associated PVT-1 translocation events have been documented (Jin et al., 2019), underpinning the reported roles of this lncRNA in oncogenesis (section Amplifications and Deletions). Additional, translocation events disturbing disease-associated lncRNA loci have been described (Figure 3). Well-documented examples are depicted below.

#### Growth Arrest Specific Transcript 5

Growth arrest specific transcript 5 (GAS5) was originally identified as a serum-starvation induced transcript (Schneider et al., 1988), which functions as a decoy of the glucocorticoid receptor (Kino et al., 2010). Fusion of the GAS5 and the BCL6 oncogene as the consequence of a *t*(1;3)(q25;q27) translocation was reported in a patient with B-cell lymphoma (Nakamura et al., 2008). While there is presently no indication for a broader relevance of this fusion event in cancer, GAS5 has in the meantime been confirmed as a transcript with broad implications in oncogenesis and with prognostic potential for various cancers (Ji et al., 2019).

#### DIRC3

Another lncRNA disrupted by a documented cancer-relevant translocation event is DIRC3. In a family with renal cell cancer, the t(2;3)(q35;q21) translocation was identified, and several genes were mapped to the chromosomal break-point (Bodmer et al., 2002, 2003). The translocation results in a fusion of the DIRC3 lncRNA gene with the HSPBAP1 gene, with yet to be determined mechanistic consequences. In a screen for lncRNAs targeted by the melanoma transcription factors MITF and SOX10, DIRC3 was identified as a top hit and characterized as a melanoma tumor suppressor (Coe et al., 2019). Mechanistically, DIRC3 seems to promote expression of the tumor suppressor IGFBP5 through a chromatin loop, which brings both genes into close proximity. MITF and SOX10 suppress expression of DIRC3, which potentially contributes to melanoma development (Coe et al., 2019). Of note, MITF is co-gained with lncRNA SAMMSON (see section Amplifications and Deletions), suggesting several lncRNAs to act in a common cancer-associated circuitry.

#### Disrupted in Schizophrenia 2

Besides cancer, lncRNA-affecting translocations have also been linked to psychiatric and developmental disorders. The “disrupted in schizophrenia” genes *DISC1* and *DISC2*, for instance, map to a locus on chromosome 1, where they are transcribed into opposite directions. Whereas DISC1 encodes a peptide, DISC2 is transcribed into a lncRNA molecule and overlaps with DISC1 (Figure 3). The DISC locus resides within a chromosomal breakpoint region and is disrupted by the balanced translocation t(1;11)(q42;q14.3), which co-segregates with schizophrenia and affective disorders (Millar et al., 2000; Blackwood et al., 2001). DISC1 functions as a signaling hub, which aggregates proteins involved in pathways relevant to neuronal function and psychiatric illness (Chubb et al., 2008). The molecular function of schizophrenia associated lncRNA DISC2 remains to be clarified.

#### DA125942

Brachydactyly Type E (BDE) is a genetic disorder, characterized by shortened metacarpal and metatarsal bones. Mutations, in the HOXD13 and parathyroid hormone-like hormone (PTHLH) genes have been linked to the disease (Klopocki et al., 2010). PTHLH is an important regulator of chondrogenesis, the process of cartilage formation and condensation (Stricker and Mundlos, 2011). In BDE affected individuals, balanced translocations affecting chromosome 12p were identified, which disrupt a cis-regulatory element required for normal PTHLH expression (Maass et al., 2012). This element was found to be transcribed and give rise to an lncRNA, DA12594. In normal cells, DA12594 lncRNA was found to physically associate with chromatin at the PTHLH gene and to be required for PTHLH transcription. The authors proposed that the identified translocations in BDE patients disrupt the regulatory interaction between the DA12594 lncRNA locus and the PTHLH gene, thereby causing reduced PTHLH expression and disturbed chondrogenesis (Maass et al., 2012; Figure 3).

#### LINC00237

Genetic obesity syndromes and developmental disorders may be caused by a range of different genomic aberrations, including the IPW lncRNA gene disruption in PWS (see section Amplifications and Deletions). MOMO syndrome is another rare, genetic disorder, characterized by macrosomia, obesity, macrocephaly, and ocular abnormalities, as well as mental disability (Moretti-Ferreira et al., 1993; Di Donato et al., 2012). In a patient diagnosed with MOMO syndrome, Vu et al. (2012) identified the homozygous balanced translocation t(16;20)(q21;p11.2). The breakpoint at 20p11.23 disrupted the ncRNA gene LINC00237 and thereby, the t(16;20)(q21;p11.2) translocation interfered with expression of this lncRNA (Vu et al., 2012). In summary, the molecular dissection of the t(16;20)(q21;p11.2) translocation revealed yet another lncRNA causatively linked to a human developmental disorder; the precise molecular function of LINC00237 in human cells, however, remains to be determined.

#### RMST

Another rare developmental disorder is the Kallmann syndrome (KS), which is characterized by a disturbed gonadotropin-releasing hormone (GnRH) balance, and thus delayed sexual maturation, combined with anosmia. Several gene defects resulting in GnRH deficiency could be attributed to KS. Of note, in a KS patient, the balanced translocation *t*(7;12)(q22;q24) was identified, disrupting the RMST lncRNA gene and causing reduced RMST expression in GnRH-targeted neurons. Furthermore, expression of several genes associated with the GnRH pathway was reduced (Stamou et al., 2020). RMST was previously shown to regulate neuronal differentiation by associating with hnRNPA2/B1 and the SOX2 transcription factor, thereby promoting SOX2 target gene expression (Ng et al., 2013). Together, these reports suggest that RMST disruption contributes to KS by affecting proper GnRH-dependent neuronal maturation at the chromatin level.

Additional, lncRNA genes were shown to be affected by translocation events. Lnc-NR2F1, for instance, is another lncRNA implicated in neurogenesis and involved in developmental disorders. This lncRNA was found to be disrupted by a *t*(5;12) translocation in a family with neurodevelopmental symptoms. Lnc-NR2F1 binds to chromatin and regulates neuronal genes. In the same study, additional lncRNAs were found to be recurrently mutated in patients with intellectual disability and autism spectrum disorders (Ang et al., 2019). Other lncRNAs, demarcating chromosomal translocation break points exist (Delihas, 2018) and remain to be mechanistically dissected or causally implicated in human diseases. Furthermore, altered activity of signaling pathways and transcription factors due to translocations affecting coding genes impacts the expression of disease-associated classical GENCODE lncRNAs and enhancer RNAs, thereby e.g., contributing to leukemia and drug-resistance (Teppo et al., 2016; Han et al., 2019; Ng et al., 2019). Additionally, lncRNAs may fuse to coding genes to promote their activation. An example is the documented fusion of MALAT1 to GLI1, which enhances GLI1 expression, and thus hedgehog signaling in different types of cancer (Spans et al., 2016; Graham et al., 2017; Antonescu et al., 2018). Finally, lncRNAs might actively contribute to translocation events, as exemplified by lncRNA RUNXOR. This lncRNA seems to be engaged in chromatin loops, potentially involved in RUNX1 translocations, often observed in AML (Wang et al., 2014).

Taken together, the here depicted examples suggest important contributions of lncRNA-affecting translocations to a variety of human diseases and predict that systematic mapping of disease-causative long-range alterations in lncRNA genes will further improve diagnosis and patient stratification. An example is the facilitated discrimination of Prader-Willi and MOMO syndrome by distinct genomic signatures in lncRNA gene loci. While the discrimination in this case does not necessarily require molecular diagnostics, the latter may further underpin the disease-cause and pave the way for a better understanding of obesity-related genetic diseases and therapeutic angles. It remains to be determined, whether the respective lncRNAs may serve as direct therapeutic targets for tailored therapies (see section Discussion).

## SNPs in IncRNA Loci Causally Implicated in Disease

The decryption of the human genome sequence and the increased availability of array and next-generation sequencing technologies have propelled the identification of genetic variants, associated with specific disease traits. Besides large genomic aberrations, focal amplifications and deletions, GWASs have already mapped thousands of disease-relevant SNPs (MacArthur et al., 2017). However, the clinical relevance of GWAS may be limited by the fact that individual SNPs typically account for only a small proportion of the variants underlying heritability of complex traits (Tam et al., 2019). In addition, GWAS approaches often focus on disease indicators, which are easy to quantify (e.g., the body-mass-index) and may thus underestimate disease complexity (Tam et al., 2019). On the other hand, GWAS may pinpoint molecular patho-mechanisms, which can guide the development of novel therapeutics and narrow down personalized treatment options, based on patient genotyping data.

**Figure 4:**
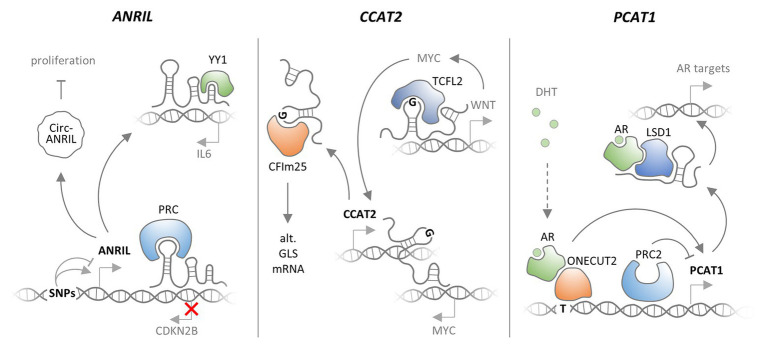
Functional consequences of SNPs affecting lncRNAs. Different SNPs in the ANRIL lncRNA gene promoter promote and repress ANRIL expression, respectively. ANRIL associates with polycomb repressor complexes (PRCs) to silence CDKN2B expression. ANRIL may also bind the YY1 transcription factor to promote IL6 expression. Finally, a circular form of ANRIL was reported to promote proliferation. ANRIL promoter SNPs may impact on these cancer- and inflammation-relevant functions of ANRIL lncRNA. CCAT2 lncRNA is transcribed from chromosome 8 and may bind to the MYC locus on the same chromosome, thereby promoting MYC expression. Alternatively, CCAT2 may bind TCFL2 to promote WNT and thereby MYC expression. A SNP was shown to elevate CCAT1 expression. The SNP also promotes binding of CCAT2 to CFIm25, thereby promoting alternative splicing of GLS mRNA and thus cancer cell proliferation. LncRNA PCAT1 is under negative control by the PRC2 complex. A prostate cancer-associated SNP in the PCAT1 promoter confers increased binding of the dihydrotestosterone (DHT) associated androgen receptor (AR), in complex with the one-cut transcription factor, which leads to increased PCAT1 levels. In complex with AR and LSD1, PCAT1 promotes expression of cancer associated genes.

Several possible scenarios can be envisioned, by which SNPs affect the functions of lncRNAs (Figures 2, 4). Disease-associated SNPs are often found in regulatory promoter elements (Hindorff et al., 2009; Maurano et al., 2012), where they may alter the binding of transcription factors, and thus impact on lncRNA expression levels. Single nucleotide variants may also be located in mature lncRNA sequences and affect protein binding motifs, e.g., required for post-transcriptional control of RNA stability. SNPs within lncRNA secondary structures, such as hairpin loops, may alter RNA folding and thereby the interaction with other biomolecules. SNPs may also affect splice-sites, thereby promoting the accumulation of alternative splice-variants with altered functionality (Figure 2). While a complete list of published SNPs within lncRNA loci would be beyond the scope and limits of the current review, the following sections cover well-characterized examples and illustrate mechanistic consequences of lncRNA SNPs in the context of human diseases.

### SNPs Affecting lncRNA Genes Implicated in Oncogenesis

Cancer remains a leading cause of death world-wide, and research over the past decade has identified a number of common cancer driver mechanisms. Since cancer may originate from a variety of different tissues and cell types, patients may profit from personalized therapies based on their cell-type specific driver mutations (Nik-Zainal et al., 2020). LncRNAs are generally more cell-type specific than mRNAs and recently, potential driver mutations in lncRNA loci have been revealed (Carlevaro-Fita et al., 2020). Thus, lncRNAs might constitute attractive candidates for prospective personalized therapies (Nguyen and Carninci, 2016). The database LincSNP 2.0 currently lists 371,647 disease-associated SNPs in lncRNA loci, of which ~11% overlap with transcription factor binding sites in lncRNA promoters (Ning et al., 2017). Among the overall disease-associations reported by the lncRNA disease database, lncRNAs most frequently associate with cancer and the same holds true for lncRNA gene polymorphisms (Bao et al., 2019). In the following, well-documented cancer-associated SNPs affecting lncRNA loci and their mechanistic consequences in the context of disease predisposition and pathogenesis are described.

#### ANRIL

Already before the rise of GWAS, deletions of the ANRIL lncRNA gene upstream of the CDKN2A and CDKN2B genes have been associated with increased risk of melanoma and neural system tumor development in several families (see section Amplifications and Deletions). The ANRIL locus contains three coding genes (*CDKN2A*, *CDKN2B*, and *ARF*) that are critical for the function of the retinoblastoma (RB) and p53 tumor suppressor networks (Sherr, 2012). ANRIL overlaps with the *ARF* and *CDKN2B* genes and is co-expressed with both genes under normal and pathologic conditions (Pasmant et al., 2007). Several GWASs have associated SNPs in this region not only with diverse types of cancer (Stacey et al., 2009; Bei et al., 2010; Turnbull et al., 2010), but also other diseases, such as coronary heart disease (Helgadottir et al., 2007; McPherson et al., 2007; Broadbent et al., 2008), or Type 2 diabetes (Diabetes Genetics Initiative of Broad Institute of Harvard and MIT, Lund University, and Novartis Institutes of BioMedical Research et al., 2007; Scott et al., 2007; Zeggini et al., 2007). By now, it has become widely accepted that SNPs in the CDKN2A and CDKN2B upstream region act predominantly through lncRNA ANRIL (Pasmant et al., 2011). In line with this view, SNPs in the CDKN2A/CDKN2B locus were reported to impact ANRIL lncRNA expression (Cunnington et al., 2010). Disease associated SNPs were reported to affect a repressive STAT1 binding site near the ANRIL locus (Harismendy et al., 2011). Derepressed ANRIL in turn negatively regulates the neighboring CDKNB gene (Yap et al., 2010; Harismendy et al., 2011). Mechanistically, ANRIL binds to CBX7, a component of the PRC1 and promotes silencing of CDKNB expression in a PRC1- and histone H3K27me-dependent manner (Yap et al., 2010). Disease SNPs or promoter deletions, negatively impacting on ANRIL expression (Cunnington et al., 2010), might relieve CDKNB repression through the same mechanism. In line with the inflammatory component in coronary artery disease, which ANRIL SNPs predispose to, ANRIL expression is elevated by pro-inflammatory stimuli, such as the cytokines interferon-gamma (IFNγ) or TNFα (Harismendy et al., 2011; Zhou et al., 2016b). Under these conditions, ANRIL was found to interact with the YY1 transcription factor to promote expression of important mediators of the inflammatory response (Zhou et al., 2016b). Thus, lncRNA ANRIL, expression of which is affected by several disease-associated SNPs, regulates gene expression both in *cis* and in *trans* at the chromatin level (Figure 4). Notably, ANRIL was also described to exist in a circularized form, which controls ribosomal RNA maturation and inhibits cell proliferation (Burd et al., 2010; Holdt et al., 2016). This suggests that ANRIL is a multifunctional lncRNA involved in both transcriptional and post-transcriptional regulations of disease-relevant cellular pathways.

#### CCAT2

Similar to ANRIL, the CCAT2 lncRNA gene, located in chromosomal region 8q24.21, has been implicated in several malignancies, and CCAT2 expression is increased in many types of cancer (Xin et al., 2017). The CCAT2 gene region contains a colorectal cancer associated SNP (rs6983267), and the risk allele was shown to promote CCAT2 overexpression (Ling et al., 2013). Mechanistically, CCAT2 binds to TCFL2 to promote expression of WNT signaling genes. CCAT2 is itself a WNT signaling target, suggesting that the lncRNA establishes a positive feed-back loop, promoting the activity of this pathway and eventually WNT-dependent MYC proto-oncogene expression (Ling et al., 2013). The CCAT2 SNP (rs6983267) region was also reported to interact with the MYC promoter (Pomerantz et al., 2009), suggesting that the CCAT2 gene might directly impact on MYC expression through an enhancer-like function. The G-allele of the CCAT2 rs6983267 SNP has also been reported to preferentially bind the CFIm25 subunit of the cleavage factor I (CFIm) complex and promote alternative splicing of Glutaminase (GLS) mRNA, to promote cancer cell metabolism and proliferation (Redis et al., 2016). Furthermore, CCAT2 transgenic mice, carrying the disease-SNP alleles develop myeloid malignancies, which can be traced back to impaired PRC2 function (Shah et al., 2018). Thus, CCAT2, similar to ANRIL appears to be a multifunctional lncRNA, involved in cancerogenesis through transcriptional and post-transcriptional mechanisms (Figure 4).

#### PCAT1

PCAT1 was identified as an lncRNA overexpressed in a subset of prostate cancer patients, where it promotes cell proliferation (Prensner et al., 2011). PCAT1 expression is under negative control by the PRC2 complex. Consequently, patients could be stratified into groups with either PCAT1 lncRNA or PRC2 protein expression (Prensner et al., 2011). A SNP in a PCAT1 enhancer region (rs7463708) causes increased binding of the androgen receptor (AR) in complex with the ONECUT2 transcription factor, and consequently promotes elevated PCAT1 expression (Guo et al., 2016). PCAT1 lncRNA was shown to interact with an AR/LSD1 complex to promote expression of androgen-stimulated genes involved in prostate cancer progression (Guo et al., 2016; Figure 4). Meanwhile, PCAT1 has been implicated in many types of cancers, and further cancer-associated PCAT1 SNPs have been identified (Yang et al., 2019). The risk variant rs72725854 (A > T) for instance, locates to a prostate-specific enhancer and promotes expression of 8q24 lncRNAs PCAT1, PRNCR1 and PVT1 through SPDEF transcription factor recruitment (Walavalkar et al., 2020). Thus, similar to ANRIL and CCAT2, PCAT1 is a major oncogenic lncRNA and potential prognostic and therapeutic cancer marker.

#### HULC

HULC was first identified as an lncRNA overexpressed in hepatocellular carcinoma (Panzitt et al., 2007). Meanwhile, HULC lncRNA levels were shown to be altered in many types of cancer, through a complex network of transcriptional and post-transcriptional control mechanisms (Yu et al., 2017). The rs7763881 SNP in the HULC locus was associated with decreased risk of hepatocellular carcinoma development in patients with persistent HBV infection (Liu et al., 2012). Likewise, in colorectal and esophageal cancer studies, the rs7763881 SNP was found to be protective (Kang et al., 2015; Shaker et al., 2017). Several mechanisms have been proposed, through which HULC impacts mRNA expression and cancerogenesis. For instance, HULC was proposed to interact with YB-1 to promote phosphorylation and thereby dissociation of YB-1 from repressed messengers, including cyclin D1 and E1 mRNAs (Li et al., 2017a). Alternatively, HULC was suggested to bind to chromatin factors coREST and hnRNPK to promote the expression of pro-inflammatory genes, such as IL6 (Chen et al., 2019b). In summary, while the association of lncRNA HULC with cancer is well-established, the precise mechanistic contribution of the rs7763881 SNP to HULC function remains to be determined.

#### CUPID1 and CUPID2

The 11q13 breast cancer risk locus contains several SNPs associated with estrogen-receptor-positive tumors. Betts et al. (2017) identified two lncRNAs, CUPID1 and CUPID2, which are transcribed from this locus in a head-to-head orientation. Transcription of both lncRNAs depends on the distal enhancer element PRE1. Importantly, the 11q13 breast cancer SNPs rs661204 and rs78540526 negatively affect the activatory PRE1 interaction with the CUPID1 promoter. Mechanistically, CUPID1 and CUPID2 locate to nuclear foci and promote DNA repair through homologous recombination but do not affect non-homologous end joining. This suggests that CUPID1 and CUPID2 are involved in the choice of the DNA repair pathway upon DNA double strand breaks (Betts et al., 2017). Thus, SNPs in the 11q13 locus likely contribute to breast cancer risk by disturbing PRE1 enhancer interaction with the shared promoter of DNA repair associated lncRNAs CUPID1 and CUPID2.

Besides ANRIL, CCAT2, PCAT1, HULC, and CUPID1/2, there are numerous other cancer-associated lncRNAs listed in the relevant databases (Table 1), several of which contain disease-associated SNPs (Ning et al., 2017; Bao et al., 2019). PTCSC3, for instance, is a tumor suppressor lncRNA located downstream of the rs944289 SNP at 14q.13.3, which predisposes to papillary thyroid carcinoma. The rs944289 SNP destroys a C/EBPα/β binding site, which reduces the expression of PTCSC3 (Jendrzejewski et al., 2012). MIR2052HG is a breast-cancer relevant lncRNA, which harbors the rs4476990 and rs3802201 SNPs. Both SNPs foster estradiol-induced, ERα-dependent expression of MIR2052HG, which in turn promotes ERα expression and stability (Ingle et al., 2016). HOTAIR is not only a non-coding RNA regulator of HOX gene expression (Rinn et al., 2007), but also implicated in oncogenesis (Shah and Sukumar, 2010). Consequently, GWASs identified several cancer associated SNPs in the HOTAIR locus (Tang and Hann, 2018). One of these SNPs locates to an intronic enhancer and alters HOTAIR expression levels (Zhang et al., 2014). Further examples of lncRNAs with cancer SNPs include linc-PINT, MEG3, GAS5, PTENP1, H19, HOTTIP, CCAT1, or the P53-regulatory lncRNA network (Ning et al., 2017; Wang et al., 2017; Minotti et al., 2018; Carlevaro-Fita et al., 2020). Many other, less well-characterized lncRNA loci overlap with cancer risk variants or interact with distant risk variant regions through chromatin loops (Moradi Marjaneh et al., 2020). An emerging aspect of cancer-associated lncRNAs, with potential relevance to personalized therapy approaches, is their role in drug resistance. Some of the above-depicted lncRNAs with cancer SNPs, deletions and amplifications, such as HOTAIR, ANRIL, or MALAT1 were implicated in the development of treatment-resistant cancer cells (Liu et al., 2020). Taking into account, patients non-coding genome sequence information could therefore improve the determination of appropriate treatment regimens. Effective utilization of this information, however, demands a robust understanding of the causality between candidate lncRNA mutations and cancerogenesis or drug resistance. Recent research on lncRNA MALAT1 and MIR122HG associated mutations illustrates the difficulties regarding the establishment auf such causality. Indel mutations in the MALAT1 and MIR122 host gene loci were identified in a pan-cancer analysis of somatic mutation hotspots (Rheinbay et al., 2020). Interestingly, MIR122 mutations were found outside of the miRNA encoded in this gene locus, and no alterations in miR-122 target-levels could be associated with this mutation, suggesting that they rather affect the miRNA host gene. Importantly, however, both MIR122 and MALAT1 indel mutations could not be confirmed as driver-mutations (Carlevaro-Fita et al., 2020; Rheinbay et al., 2020), and their roles in cancer thus remain unclear. It is therefore important, to aggregate information from diverse sources, including e.g., mutagenesis screens and mechanistic evidence, to establish causative relationships between lncRNA gene variations and cancer.

### SNPs in Inflammation-Relevant lncRNA Loci

Besides cancer, infectious, and inflammatory diseases have remained a leading human health burden and a major cause of morbidity and mortality worldwide, despite great improvements in prevention and treatment (Holmes et al., 2017). Mortality associated with fulminant inflammation during sepsis for instance has remained difficult to control with broadly acting anti-inflammatory therapeutics, such as corticosteroids (Rochwerg et al., 2018). An improved understanding of the molecular circuits driving immune-defense and inflammation is therefore required to establish more targeted therapies for inflammation-induced pathologies. Recently, several lncRNAs were implicated in the cellular circuitries controlling inflammation, and genetic evidence for a role of lncRNAs in human inflammatory diseases has been provided (Castellanos-Rubio and Ghosh, 2019). This section depicts well-documented examples.

#### Lnc13

Celiac disease is a chronic illness, in which exposure to dietary gluten triggers inflammation in the intestine, often also accompanied by systemic symptoms (Ludvigsson et al., 2013; Tye-Din et al., 2018). Celiac disease affects genetically predisposed individuals, and risk-associated SNPs have been identified. The rs917997 SNP downstream of the IL18RAP locus for instance was reported to predispose to celiac disease (Hunt et al., 2008; Koskinen et al., 2009), the underlying mechanism however had remained unknown. In 2016, Castellanos-Rubio et al. (2016) reported an lncRNA, lnc13, which originates from the IL18RAP locus and harbors the rs917997 SNP. Lnc13 represses inflammatory gene transcription by associating with hnRNPD in the nucleus and fostering promoter-binding of HDAC1 (Castellanos-Rubio et al., 2016). Expression levels of lnc13 were found to be reduced in celiac disease patients, which might contribute to increased inflammatory gene expression. Moreover, the rs917997 SNP in lnc13 was found to weaken the interaction of lnc13 with its interaction partner hnRNPD (Castellanos-Rubio et al., 2016). In summary, the celiac disease predisposing SNP rs917997 SNP seems to impact the function of an inflammation-limiting lncRNA by disturbing its association with nuclear protein machineries.

#### IFNG-AS1

The Tmepvp3 locus has long been known as a Theiler’s virus susceptibility locus in mice, without a mechanistic explanation. Eventually, a lncRNA (IFNG-AS1, a.k.a. Tmevpg1 or NeST), mapping to the Tmevpg3 locus was shown to control expression of the nearby IFNγ gene, and a human homolog was identified (Vigneau et al., 2003). IFNγ is a major cytokine produced preferentially by T- and NK-cells and involved in Th1 responses and antibacterial defense. IFNG-AS is highly expressed in Th1-polarized T-cells, where it depends on the T-bet transcription factor and positively regulates IFNγ expression in cis (Collier et al., 2012; Padua et al., 2016; Petermann et al., 2019). The IFNG-AS gene overlaps with the IBD susceptibility SNP rs7134599 and was identified as the only out of several IBD SNP associated lncRNAs increased in ulcerative colitis patients (Mirza et al., 2015; Padua et al., 2016). Furthermore, elevated circulating IFNG-AS1 levels correlate with coronary artery disease severity (Xu and Shao, 2018). IFNG-AS1 was also shown to protect from severe systemic *Salmonella enterica* infection outcomes in mice, by promoting IFNγ expression (Gomez et al., 2013). Mechanistically, in the same study, IFNG-AS1 was shown to interact with the WDR5 histone-methyltransferase in the nucleus, to promote the deposition of activating histone methylation marks at the IFNγ locus. In summary, IFNG-AS1 is an IFNγ promoting chromatin-regulatory lncRNA, genetically associated with inflammation-linked human diseases.

#### LncRNA CCR5AS

The CCR5 chemokine receptor on the surface of T cells serves as a major HIV co-receptor during cell attachment and entry. Consequently, gene variants causing loss of CCR5 surface expression were found to confer resistance to HIV infection (McLaren and Carrington, 2015). GWASs have detected several SNPs, which impact on HIV infection (McLaren and Carrington, 2015). One of the top-ranking SNPs (rs1015164) on chromosome 3 maps down-stream of the CCRL2 locus, near an annotated antisense transcript (CCR5AS or RP11-24F11.2), which overlaps with the CCR5 gene body (McLaren et al., 2015; Kulkarni et al., 2019). The rs1015164 SNP marks an ATF1 binding site and enhances expression of CCR5AS. Knockdown of CCR5AS was found to reduce CCR5 mRNA abundance (Kulkarni et al., 2019). Mechanistically, CCR5AS was proposed to decoy RNA binding protein RALY, thereby enhancing CCR5 mRNA stability and consequently susceptibility to HIV infection (Kulkarni et al., 2019). Thus, lncRNA CCR5AS seems to constitute a central player in HIV resistance due to nucleotide variations in the CCR5 gene locus.

Besides the above depicted well-documented examples, further lncRNAs have been genetically linked to inflammation and infection, including e.g., streptococcal bacteremia associated lncRNA AC011288.2 (Kenyan Bacteraemia Study Group et al., 2016), but are still to be independently confirmed or explained mechanistically. Furthermore, lncRNAs with cancer-associated SNPs, such as NEAT1, Gas5, or ANRIL have been linked to inflammation (Castellanos-Rubio and Ghosh, 2019; Chen et al., 2019a). Additionally, increasing biochemical evidence suggests that many more lncRNAs participate in the cellular circuitries controlling inflammatory gene expression, including e.g., PACER, lincRNA-Cox2, or lincRNA-EPS (Chen et al., 2019a). Besides these established inflammation regulators, we recently determined dozens of so far uncharacterized lncRNAs, which are significantly up- or down-regulated upon bacterial infection or immune-activation of primary human cells (Aznaourova et al., 2020; Schulte et al., 2020). One of these lncRNAs, MaIL1, was identified by us as an essential component of the TLR4-TRIF immune-signaling pathway, mediating interferon expression in response to infectious agents. MaIL1 levels were increased in bronchoalveolar lavage fluid from patients with pulmonary infections (Aznaourova et al., 2020). Thus, increasing genetic and biochemical evidence suggests a vital participation of lncRNAs in human immunity, with potential relevance to patient stratification and prospective personalized therapies, as further discussed below (section Discussion).

### SNPs in Other Disease-Associated lncRNA Loci

Many prevalent diseases involve an inflammatory component, including e.g., cardiovascular pathologies or cancer. Thus, it is not surprising, that several lncRNAs with disease-associated polymorphisms were implicated at the intersection of these three leading causes of death. Examples include NEAT1, Gas5, or ANRIL (see above), but also H19, LINC00305 and various other lncRNAs (Giral et al., 2018; Minotti et al., 2018; Ramsuran et al., 2018; Castellanos-Rubio and Ghosh, 2019). Besides these three major human health burdens, lncRNA SNPs have also been associated with rather rare disorders, such as HELLP (hemolysis, elevated liver enzymes, and low platelets). LncRNA H19, LINC00305, and Linc-HELLP are portrayed below as examples for lncRNAs linked to cardiovascular and rare diseases by GWAS.

#### H19

H19 was one of the first lncRNAs to be discovered and has been implicated in diseases, such as atherosclerosis (Han et al., 1996), though its non-coding nature was only recognized years after the H19 cDNA was first cloned. Originally, H19 was discovered as an RNA induced early during murine embryogenesis and muscle cell differentiation (Pachnis et al., 1988), which is reactivated during vascular smooth muscle injury (Kim et al., 1994). Meanwhile, several SNPs in the H19 lncRNA locus have been linked to increased risk of coronary artery disease (Gao et al., 2015) and predisposition to elevated blood-pressure (Tragante et al., 2014; Sung et al., 2018). Furthermore, H19 SNPs have been associated with several types of cancer (Verhaegh et al., 2008; Li et al., 2018a) and with elevated serum levels of H19 (Yang et al., 2015). Mechanistically, H19 was proposed to serve as a let-7 microRNA sponge in the cytosol (Kallen et al., 2013). On the other hand, H19 was reported to interact with chromatin factors in the nucleus to regulate gene expression (Monnier et al., 2013). Thus, while the role of lncRNA H19 in cardiovascular diseases is well-established, the precise molecular mode of H19 function, explaining the GWAS-identified disease predispositions is still under debate.

#### MIAT

MIAT is another lncRNA with well-documented roles in cardiovascular diseases and cancer. Originally identified as a myocardial infarction risk locus (Ishii et al., 2006), MIAT was soon suggested as a cardiovascular disease biomarker (Qu et al., 2017; Zhu et al., 2018). The rs2331291 (C > T) SNP in the fifth exon of MIAT was not only shown to be significantly associated with myocardial infarction, but also to promote MIAT1 expression, as well as its interaction with nuclear proteins (Ishii et al., 2006). The murine MIAT1 orthologue was found to interact with the nuclear matrix (Sone et al., 2007) and associate with splice factor SF1 *via* a conserved repeat motif, possibly to establish local nuclear splice-regulatory compartments (Tsuiji et al., 2011; Ishizuka et al., 2014). While the precise molecular contributions of MIAT to disease remain to be determined, the studies available so far suggest this lncRNA as a promising molecular prognosis marker among an array of further cardiovascular disease-relevant lncRNAs (Lozano-Vidal et al., 2019).

#### LINC00305

Besides H19 and MIAT, LINC00305 is another lncRNA with a potential role in cardiovascular diseases. A SNP in an intronic segment of the LINC00305 gene (rs2850711) had been associated with atherosclerosis, which prompted Zhang et al. (2017b) to investigate the expression levels of this previously uncharacterized lncRNA in a patient cohort. LINC00305 expression was significantly increased in atherosclerotic plaques compared to normal artery samples (Zhang et al., 2017b). Furthermore, LINC00305 levels were higher in monocytes compared to endothelial and aortic smooth muscle cells. In monocytes, LINC00305 was found to promote inflammatory gene expression, probably involving NFκB activation in a lipocalin-1 interacting membrane receptor (LIMR) and aryl-hydrocarbon receptor repressor (AHRR) dependent manner (Zhang et al., 2017b). In endothelial cells, LINC00305 was furthermore assigned a pro-apoptotic function (Zhang et al., 2017a). Increased expression of LINC00305 was also observed in serum of rheumatoid arthritis (RA) patients. Furthermore, RA patients carrying the rs2850711 polymorphism had significantly elevated LINC00305 and pro-inflammatory marker expression (Wahba et al., 2020). Together, these reports suggest that an intronic SNP in the LINC00305 gene predisposes to atherosclerosis and arthritis by increasing expression of apoptosis and inflammation promoting lncRNA LINC00305.

#### Linc-HELLP

HELLP (hemolysis, elevated liver enzymes, and low platelets) is a life-threatening pregnancy-associated syndrome, which entails initial placental dysfunction and eventual systemic maternal symptoms. An intergenic HELLP locus with several disease-associated SNPs on chromosome region 12q23.2 was identified, which gives rise to an lncRNA (linc-HELLP) and is flanked by the PMCH and IGF1 genes (van Dijk et al., 2012). Knockdown and RNA-Seq analysis of linc-HELLP in trophoblast cells suggested an involvement in cell survival and cell-cycle progression (van Dijk et al., 2012). Familial mutations in Linc-HELP negatively affect trophoblast differentiation and alter the binding of protein interaction partners involved in splicing and ribosomal function (van Dijk et al., 2015). Thus, linc-HELLP constitutes another example of an lncRNA involved in critical cellular pathways and genetically associated with a severe human disease.

Besides the well-documented examples summarized in this review, many other disease-relevant lncRNA SNPs have been identified and are listed in the relevant databases (Table 1). In the context of cardiovascular diseases, for instance, SNPs affecting the well-studied lncRNA ANRIL were pointed out (Lozano-Vidal et al., 2019). Furthermore, aberrations in non-coding genome regions corresponding to transcribed enhancers potentially contribute to human diseases (Isoda et al., 2019). A closer investigation of mutations and polymorphisms affecting the transcription or function of non-coding RNAs generated from such regulatory elements might contribute additional insights into the roles of non-coding genome alterations in human diseases (see Discussion). The majority of disease-associated SNPs, however, are still awaiting to be independently reproduced or explained mechanistically. Furthermore, even for well-documented disease-associations of lncRNA SNPs, such as the rs2331291 SNP in the MIAT locus, the aspect of causality between nucleotide variants and disease demands further investigation, as discussed below. Despite the still many open questions, however, a growing body of literature suggests that lncRNA gene loci, once regarded as evolutionary remnants and “junk DNA,” fulfill critical functions in human physiology and disease, and must be considered in next-gen diagnostics and personalized medicine approaches, similar to protein-coding genes.

## Discussion

Despite increasing evidence for an important role of lncRNAs in human diseases and their emerging application as biomarkers, their routine clinical implementation has not yet been achieved and only a fraction of the ~20,000 annotated lncRNAs has been studied to date. One reason might be that lncRNAs have not been included in large diagnostic studies for long, although disease-associated mutations affecting lncRNAs, such as ANRIL or IPW (see above) were known years before the release of comprehensive lncRNA annotations. Circumstances that might have delayed the inclusion of many lncRNAs in clinical cohort data analysis are the still provisional annotation status of many lncRNAs, their functional heterogeneity and the lack of approved pharmacological approaches to harness their therapeutic potential. While numerous recent publications have independently validated many annotated lncRNAs as non-coding transcripts with cell-type and condition-specific expression patterns, their molecular functions in diseases often remain unclear. Different from other classes of RNA, such as mRNAs, snRNAs, or miRNAs, lncRNAs do not seem to interact with a common protein-machinery (Aznaourova et al., 2020), which may render their investigation challenging. Recent studies have, however, substantially improved our understanding of lncRNA subcellular localization, coding-capacity, and interaction with protein-complexes (Carlevaro-Fita and Johnson, 2019; Aznaourova et al., 2020; Chen et al., 2020a), which will contribute to improved annotations and mechanistic models of lncRNA functions in disease. To promote the consideration of this lncRNA knowledge in clinical studies, better integration into medical NGS analysis workflows seems necessary. This demands not only the establishment of well-curated lncRNA knowledge databases but also of clinical informatics units as well as targeted training of young medical informatics staff. As a result of such measures, the advent of NGS in the clinics should increasingly accelerate our understanding of lncRNA functions and mutations in human diseases and eventually improve molecular diagnostics.

While research into the functions of lncRNAs in human diseases is still in its infancy, several lncRNAs have repeatedly been described as prognostic and risk stratification markers. Expression of PVT1 and FAL1, for instance, associates with advanced TNM stage in different types of cancer (Hu et al., 2014; Lu et al., 2017; Martini et al., 2017; Ni et al., 2017). Furthermore, prostate cancer patients could be stratified into distinct groups expressing either high levels of PRC 2 components or of the proliferation-promoting lncRNA PCAT1 (Prensner et al., 2011). Besides PCAT1, several other lncRNAs have prognostic potential in prostate cancer (Arriaga-Canon et al., 2018). LncRNA PCA3 was even employed as a prostate cancer marker in a liquid biopsy test, though the test specificity remains debated (Morgan, 2019). Several of these lncRNAs contain disease-associated SNPs (see above), further suggesting their utility in molecular diagnostics. For example, it is conceivable that disease-associated lncRNA gene variants will be used in the future to forecast the success of cancer therapy. In fact, several lncRNAs affected by cancer-relevant SNPs or amplifications and deletions, including H19, PVT1,or ANRIL have been implicated in cancer drug resistance, as extensively reviewed elsewhere (Campos-Parra et al., 2018; Liu et al., 2020; Peng et al., 2020; Yuan et al., 2020). To solidify the clinical utility of these lncRNAs as diagnostic and prognostic markers for defined patient groups, larger cohorts and replication studies are desirable. In the case of lncRNA RMRP, such replication attempts could already pinpoint the occurrence of promoter mutations specifically to breast cancer patients (Rheinbay et al., 2017; Son et al., 2019). Other replication studies could causally link mutations affecting lncRNAs, such as RMRP, NEAT1, or LINC-PINT, to cancer (Rheinbay et al., 2017; Carlevaro-Fita et al., 2020). Such studies represent an important pillar of the current consolidation of medical lncRNA knowledge, which might eventually lead to improved molecular diagnostics and personalized therapies.

The increasing number of studies on disease-associated variations in lncRNA loci raises the question of potential advantages of lncRNAs as clinical biomarkers and targets, compared to established protein markers. Generally, lncRNAs seem to be more tissue and cell type specific than mRNAs (Cabili et al., 2011), which predestines them e.g., for use in liquid biopsy approaches. Effective clinical utilization of lncRNA knowledge thus demands the integration of data from sources, such as tissue expression catalogs and single-cell RNA-Seq studies of diseased and healthy tissue. Well-curated knowledge databases aggregating genetic and clinical study data, biochemical insights, and expression specificity information might pinpoint highly cell-type specific lncRNAs both as novel biomarkers and as prospective targets for precise pharmacological interventions in affected tissues and organ-systems. Another advantage of lncRNA- over protein-biomarkers is that with lncRNAs, functional biomolecules and their mutations can be directly quantified by sensitive, rapid, and cost-effective PCR- or NGS-based approaches, whereas mRNA levels, as intermediates, often do not reflect protein expression at the time-point of sample collection (Liu et al., 2016) and in the case of direct protein quantifications no information on mutations is provided. Irrespective of the potential advantages of lncRNAs over proteins as biomarkers, personalized medicine approaches based on whole genome profiling may only profit from a refined understanding of disease-associated driver and passenger mutations, taking into account the long overlooked mutations in lncRNA gene regions.

The continuous improvement of human transcriptome annotations has brought further classes of non-coding RNA into the focus of genetic studies. Besides small non-coding RNAs, such as snoRNAs (Deogharia and Majumder, 2018) or microRNAs (Hrdlickova et al., 2014), genetic variation in non-coding RNAs generated from enhancer regions has received particular attention. Enhancers are typically marked by H3K4me1 signatures and by recruitment of co-activators of transcription, such as p300/CBP. Many enhancer regions in addition recruit RNAPolII and generate eRNAs, which differ from GENCODE lncRNAs in that they are typically unspliced and non-poly-adenylated (Kim et al., 2015). Besides eRNAs, RNAPolII generates lncRNAs, transcribed from enhancer regions or recruited to super-enhancers, which are mostly tissue-specific enhancer clusters, bound to Mediator (Gardini and Shiekhattar, 2015; Soibam, 2017). NcRNA transcription from such regions plays an important role in chromatin organization and enhancer-promoter communication. For instance, ncRNAs generated from enhancer regions can assist in the recruitment of factors such as CTCF or mediator, thereby contributing to chromatin loop formation (Gil and Ulitsky, 2020). Importantly, the act of transcription itself can exert important regulatory functions by affecting chromatin modification and accessibility (Ali and Grote, 2020). The act of transcription of non-coding RNA ThymoD, for instance, promotes CTCF binding site demethylation and thus formation of a chromatin-loop juxtaposing the Bcl11b enhancer and promoter, independent of the final RNA product (Isoda et al., 2017). In line with the role of Bcl11b in T cell commitment, disruption of ThymoD transcription in mice results in lymphoid malignancies (Isoda et al., 2017). In line with the critical roles of enhancers in the orchestration of gene expression in and outside of insulated chromatin domains [topologically associating domains (TADs); Fanucchi and Mhlanga, 2017], nucleotide variants and disruptions affecting enhancers, and potentially the transcription of eRNAs and lncRNAs generated from these regions, have been implicated in various diseases. This ranges from autoimmune diseases to mental disorders and cancer (Farh et al., 2015; Javierre et al., 2016; Teppo et al., 2016; Ren et al., 2017; Hauberg et al., 2019; Isoda et al., 2019; Lewis et al., 2019; Chen et al., 2020b; Yamagata et al., 2020). Thus, beyond lncRNAs encoded within coding gene regions or seemingly empty genomic space, genetic variation in transcribed regulatory DNA units, such as enhancers, contributes to human diseases. This indicates that the well-documented lncRNA gene polymorphisms and mutations summarized in the present review and in the relevant databases (Table 1) represent only a fraction of the aberrations in the non-coding genome contributing to disease.

As a result of the many recent reports on disease-relevant functions of human ncRNAs, their potential value as therapeutic targets is increasingly being recognized. mRNA-targeting antisense oligonucleotide drugs are arriving in the clinics (Shen and Corey, 2018), which makes targeting of lncRNAs seem feasible. Such RNA inhibitors are typically composed of synthetic nucleic acid mimics, such as locked nucleic acid (LNA), peptide nucleic acid (PNA), or phosphorodiamidate morpholino (PMO), which base-pair with RNA. Cellular delivery may be achieved through cell-penetrating peptides or packaging into lipid nanoparticles (Juliano, 2016; Nan and Zhang, 2018). The emerging clinical use of such RNA inhibitor technologies suggests that an improved understanding and extensive cataloguing of ncRNA circuitries, altered during human diseases, may not only reveal valuable biomarkers and improve genome-wide diagnostics approaches but also open new therapeutic avenues.

Taken together, the advent of next-generation sequencing (NGS) technologies in clinical research and diagnostics has made it increasingly feasible to pinpoint non-coding gene variants and disease markers in a highly parallelized manner, and new chemical approaches allow to manipulate RNA levels therapeutically *in vivo*. Both will improve our understanding of the implications of the emerging class of lncRNAs in disease onset and progression. Currently, replication attempts with larger cohorts, and an improved understanding of the molecular functions of lncRNA markers seem necessary before the potential of lncRNAs in personalized medicine can be fully harnessed. However, continuous progress in the mechanistic understanding of lncRNAs in disease (DiStefano, 2018) and ambitious sequencing projects, such as the 100,000 Genomes Project (Sivapalaratnam and Bioresource, 2018) promise a further refinement of our knowledge about coding and non-coding disease-associated genomic alterations in the near future. Although it may still take years to achieve a comprehensive, clinically relevant understanding of the disease-contributions of lncRNAs, whole-genome analysis of a patients coding and non-coding genome and transcriptome might eventually become part of the clinical routine, much like X-rays. Significant advances in molecular diagnostics, patient stratification, and our understanding of and intervention in molecular patho-mechanisms are to be expected.

## Author Contributions

MA and NS screened, categorized, and summarized published literature. LS screened and categorized literature, wrote the manuscript draft, and prepared the figures. MA, NS, and BS proofread, corrected, and amended the manuscript draft. All authors contributed to the article and approved the submitted version.

### Conflict of Interest

The authors declare that the research was conducted in the absence of any commercial or financial relationships that could be construed as a potential conflict of interest.
